# Higher platelet distribution width predicts poor prognosis in laryngeal cancer

**DOI:** 10.18632/oncotarget.18306

**Published:** 2017-05-30

**Authors:** Huan Zhang, Li Liu, Shuang Fu, Yan-Song Liu, Changsong Wang, Tiemin Liu, Zhi-Ping Liu, Rui-Tao Wang, Kai-Jiang Yu

**Affiliations:** ^1^ Department of Internal Medicine, Harbin Medical University Cancer Hospital, Harbin Medical University, Harbin, Heilongjiang, 150081, China; ^2^ Department of Histology and Embryology, Harbin Medical University, Harbin, Heilongjiang, 150081, China; ^3^ Department of Intensive Care Unit, Harbin Medical University Cancer Hospital, Harbin Medical University, Harbin, Heilongjiang, 150081, China; ^4^ Division of Hypothalamic Research, Department of Internal Medicine, University of Texas Southwestern Medical Center, Dallas, TX, 75390, USA; ^5^ Departments of Internal Medicine and Molecular Biology, University of Texas Southwestern Medical Center, Dallas, TX, 75390, USA; ^6^ Heilongjiang Academy of Medical Science, Harbin, Heilongjiang, 150081, China

**Keywords:** laryngeal cancer, platelet distribution width, prognosis, survival

## Abstract

**Background:**

Activated platelets promote cancer progression and metastasis. However, the prognostic value of platelet indices in laryngeal cancer remains poorly understood. The purpose of this study was to investigate the predictive significance of platelet indices in laryngeal cancer.

**Results:**

Of the 241 patients, high platelet distribution width (PDW) levels were observed in 116 (48.1 %) patients. In the Kaplan-Meier analysis, increased PDW was significantly associated with a poorer overall survival (p < 0.001). In the multivariate Cox model, PDW was an independent prognostic index for overall survival (HR=4.381, 95% CI=2.313-8.298, P < 0.001).

**Method:**

The retrospective study included 241 consecutive patients with laryngeal cancer between January 2009 and December 2009. The relationships between PDW and clinicopathological characteristics were analyzed. Kaplan-Meier method and Cox regression were used to evaluate the prognostic impact of PDW.

**Conclusions:**

Elevated PDW might be a novel prognostic marker in laryngeal cancer.

## INTRODUCTION

Laryngeal cancer represents the most common malignancy of the head and neck worldwide. Despite the improvement of the therapeutic techniques, some patients still recur after treatment. Therefore, it is of great importance to look for appropriate and effective prognostic markers in laryngeal cancer.

Platelets play a pivotal role in cancer progression and metastasis [1]. There is emerging evidence to suggest that platelets mediate tumor cell growth, angiogenesis, and dissemination [2]. Increased platelets are correlated with a decrease in overall survival and poorer prognosis in various types of cancer, including pancreatic cancer, gastric cancer, colorectal cancer, endometrial cancer, and ovarian cancer [3–7]. However, platelet count is determined by the balance between the rate of production and consumption of platelets. A normal platelet count could conceal the presence of highly hypercoagulative and pro-inflammatory cancer phenotypes in the presence of efficient compensatory mechanisms [8].

Mean platelet volume (MPV), the most commonly used measure of platelet size, is a surrogate marker of platelet activation [9]. Altered MPV levels were found in gastric cancer, ovarian cancer, lung cancer, colon cancer, and breast cancer [10–13]. Platelet distribution width (PDW), another platelet index, indicates variation in platelet size [14]. However, its clinical implications in laryngeal cancer remain unclear. In the present study, therefore, we aimed to investigate MPV and PDW levels in patients with laryngeal cancer and evaluated the effects of MPV and PDW levels on pathological parameters and clinical outcome.

## RESULTS

A total of 241 patients were enrolled in this study between Jan, 2009 and Dec, 2009. The mean age was 57.8 ± 8.5 years (range 37-80). There were 197 men and 44 women. The median observation period was 60 months. There were 190 patients who were alive at the last clinical follow-up. Finally, there were 51 cancer-related deaths at the time of the last follow-up.

**Figure 1:**
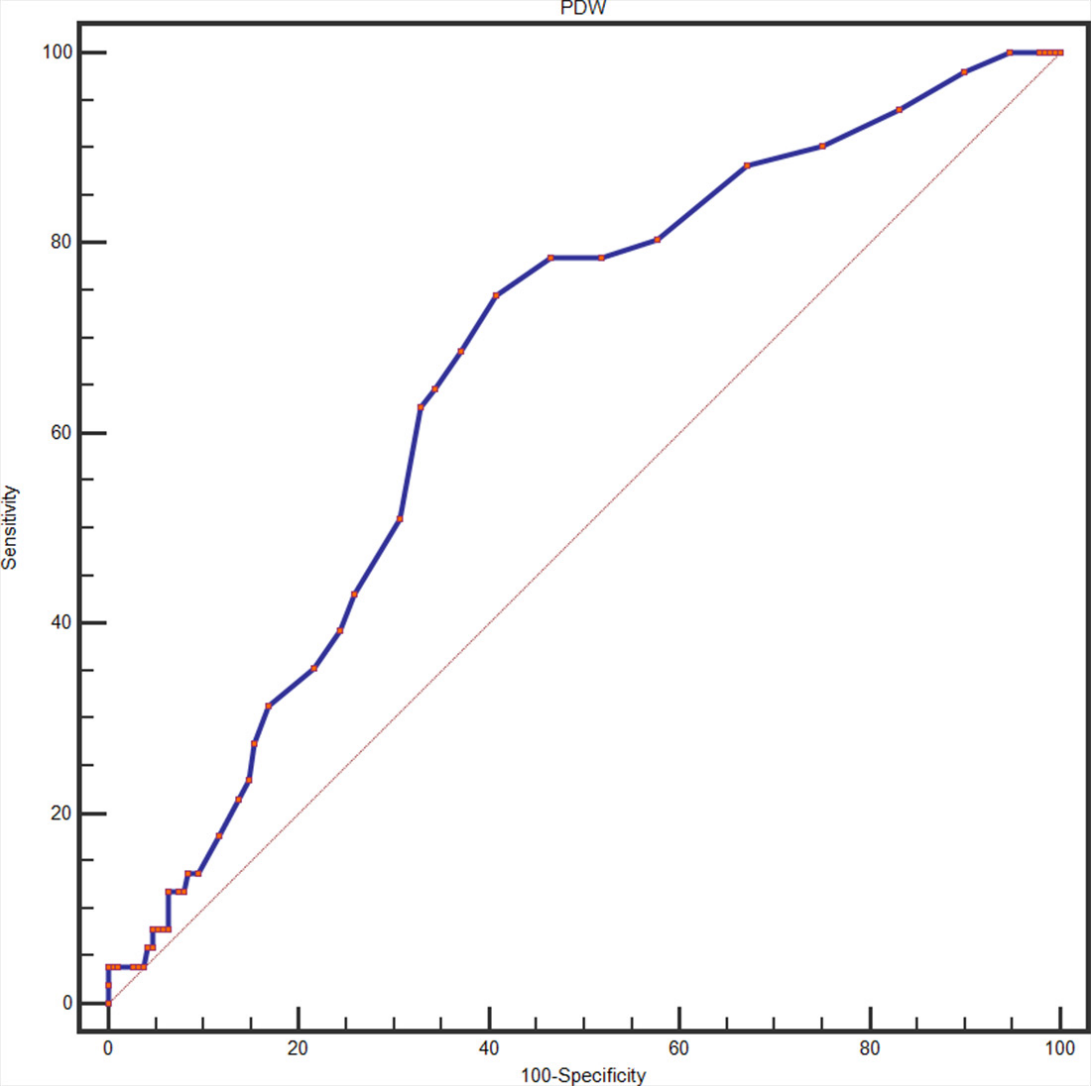
Optimized cut-off was determined for PDW using standard ROC curve analysis

A ROC curve for OS prediction was plotted to verify the optimal cut-off value for PDW, which was 16.7 % (Figure 1). It demonstrated that PDW predicts cancer prognosis with a sensitivity of 74.5 % and a specificity of 59.3 % (AUC = 0.667, 95 % CI: 0.603-0.726, p < 0.001). Then, patients were divided into 2 groups: patients with PDW ≤ 16.7 % and patients with PDW > 16.7 %. There were 125 (51.9 %) patients with PDW ≤ 16.7 % and 116 (48.1 %) patients with PDW > 16.7 %.

The relationships between PDW and clinical characteristics were shown in Table 1 and Table 2. No significant differences were found between the groups with regard to age, gender, smoking, tumor site, tumor differentiation, tumor depth, lymph node metastasis and tumor stage.

**Table 1:** Baseline characteristics of patients with laryngeal cancer according to PDW levels

Variables	Total	PDW ≤ 16.7	PDW > 16.7	P value
	n (%)	n (%)	n (%)	
Age (years)				0.907
< 60	157 (65.1)	81 (64.8)	76 (65.5)	
≥ 60	84 (34.9)	44 (35.2)	40 (34.5)	
Gender				0.467
Male	197 (81.7)	100 (80.0)	97 (83.6)	
Female	44 (18.3)	25 (20.0)	19 (16.4)	
Smoking				0.676
Nonsmokers	33 (13.7)	16 (12.8)	17 (14.7)	
Current smokers	208 (86.3)	109 (87.2)	99 (85.3)	
Tumor site				0.934
Supraglottic	65 (27.0)	34 (27.2)	31 (26.7)	
Glottic/subglottic	176 (73.0)	91 (72.8)	85 (73.3)	
Tumor differentiation				0.673
Well/moderate	206 (85.5)	108 (86.4)	98 (84.5)	
Poor	35 (14.5)	17 (13.6)	18 (15.5)	
Tumor depth				0.172
T1+T2	168 (69.7)	92 (73.6)	76 (65.5)	
T3+T4	73 (30.3)	33 (26.4)	40 (34.5)	
Lymph node metastasis				0.827
Absent	196 (81.3)	101 (80.8)	95 (81.9)	
Present	45 (18.7)	24 (19.2)	21 (18.1)	
Tumor stage				0.216
I/II	157 (65.1)	86 (68.8)	71 (61.2)	
III/IV	84 (34.9)	39 (31.2)	45 (38.8)	

PDW: platelet distribution width.

**Table 2:** Baseline characteristics of patients with laryngeal cancer according to PDW levels

Variables	PDW ≤ 16.7	PDW > 16.7	P value
Age (years)	57.6 (8.3)	58.0 (8.7)	0.670
Gender (male, %)	100 (80.0)	97 (83.6)	0.467
Smoker (n, %)	109 (87.2)	99 (85.3)	0.676
Drinking (n, %)	59 (47.2)	62 (53.4)	0.332
BMI (kg/m^2^)	22.4(2.9)	22.3 (4.6)	0.929
FPG (mmol/L)	5.20 (4.80-5.70)	5.10 (4.80-5.80)	0.686
Albumin (g/L)	43.4 (3.7)	43.0 (5.4)	0.530
WBC (×10^9^/L)	6.93 (1.72)	7.34 (2.31)	0.117
Hemoglobin (g/dl)	145.5 (15.9)	145.0 (15.2)	0.824
Platelet count (×10^9^/L)	225.2 (56.8)	219.9 (66.0)	0.507
MPV (fL)	8.5 (0.9)	9.2 (1.7)	< 0.001

Data are expressed as means (SD) or median (IQR). BMI: body mass index; FPG: fasting plasma glucose; WBC: white blood cell; MPV: mean platelet volume. Abbreviations: see to Table 1.

**Figure 2:**
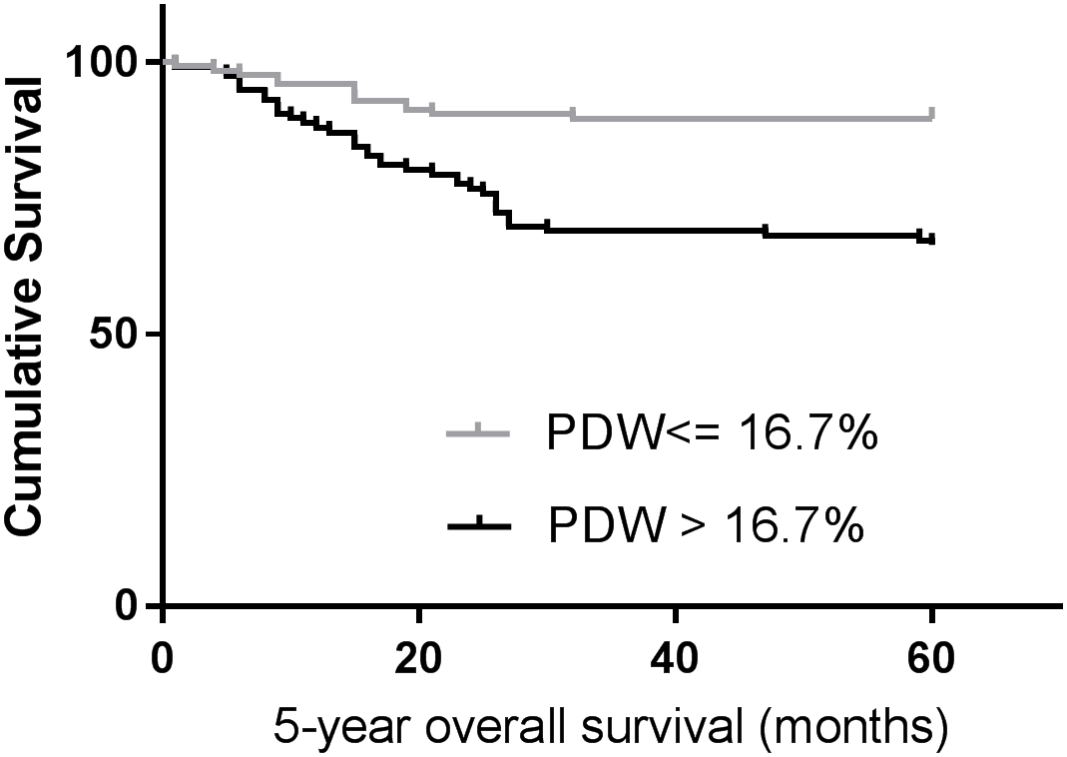
Kaplan–Meier analysis of overall survival in patients with laryngeal cancer

We used Kaplan-Meier survival analysis to evaluate the clinical outcomes between different subgroups divided by PDW levels. As presented in Figure 2, patients in high PDW levels had a worse 5-year OS (67.2 % vs. 89.6 %, P < 0.001) than those in low PDW levels. We further undertook univariate and multivariate analysis to make sure whether PDW is an independent predictor for cancer prognosis. As summarized in Table 3, PDW was strongly associated with clinical outcomes (P < 0.001) in univariate analysis. Also, age (categorical variable), platelet count, MPV (categorical variable), tumor site, tumor differentiation and tumor stage were statistically significant in univariate analysis. We put those parameters in multivariate analysis (Table 4) and confirmed that PDW still was predictable for cancer outcomes (P < 0.001). Patients with PDW > 16.7 % had a hazard ratio (HR) of 4.381 [95% confidence interval (CI): 2.313-8.298, P < 0.001] for OS.

**Table 3:** Univariate analysis of overall survival in patients with laryngeal cancer

	Hazard ratio	95% CI	*P*-value
Age (years) (≥ 60 versus < 60)	1.763	1.017–3.055	0.043
Gender (male versus female)	0.953	0.464–1.958	0.896
Smoker (yes versus no)	1.402	0.683–2.881	0.357
Drinking (yes versus no)	0.841	0.485–1.457	0.536
BMI (kg/m^2^)	0.954	0.900–1.011	0.111
FPG (mmol/L)	0.909	0.734–1.127	0.385
Albumin (g/L)	0.976	0.922–1.032	0.394
WBC (×10^9^/L)	0.976	0.847–1.124	0.736
Hemoglobin (g/dl)	0.994	0.979–1.010	0.478
Platelet count (×10^9^/L)	1.007	1.003–1.011	0.001
MPV (fL) (> 9.3 versus ≤ 9.3)	0.535	0.261–1.098	0.088
PDW (%) (> 16.7 versus ≤ 16.7)	3.515	1.872–6.601	< 0.001
Tumor site (supraglottic versus glottic/subglottic)	2.214	1.271–3.854	0.005
Tumor differentiation (poor versus moderate/well)	3.623	1.439–9.120	0.006
Tumor depth (T3+T4 versus T1+T2)	1.252	0.944–1.661	0.118
Lymph node metastasis (present versus absent)	1.138	0.784–1.652	0.495
Tumor stage (III/IV versus I/II)	1.740	1.004–3.015	0.049

Abbreviations: see to Table 1 and Table 2.

**Table 4:** Multivariate analysis of overall survival in patients with laryngeal cancer

	Hazard ratio	95% CI	*P*-value
Age (years) (≥ 60 versus < 60)	2.142	1.218–3.765	0.008
Platelet count (×10^9^/L)	1.006	1.002–1.011	0.002
MPV (fL) (> 9.3 versus ≤ 9.3)	0.506	0.239–1.071	0.075
PDW (%) (> 16.7 versus ≤ 16.7)	4.381	2.313–8.298	< 0.001
Tumor site (supraglottic versus glottic/subglottic)	1.936	1.094–3.426	0.023
Tumor differentiation (poor versus moderate/well)	3.044	1.197–7.744	0.019
Tumor stage (III/IV versus I/II)	1.609	0.923–2.804	0.093

Variables that showed a p-value < 0.10 in univariate analysis were included in a multivariate Cox proportional hazards regression model. CI: confidence interval. Abbreviations: see to Table 1 and Table 2.

## DISCUSSION

This study found that PDW is correlated with patient's survival and is an independent risk factor for prognosis in laryngeal cancer.

Despite recent interest in clinical implications of activated platelets in the setting of cancer, the scope of available data is still limited by the type of malignancy and clinical outcomes studied. Thrombocytosis is associated with reduced survival in patients with several types of malignancies, such as lung cancer, ovary cancer, endometrium cancer, rectum cancer, kidney cancer, stomach cancer, pancreas cancer, and breast cancer. Increased platelets foster cancer progression and metastasis by shielding circulating tumor cells from immune surveillance and killing [15].

The molecular mechanism to explain the association between PDW and survival in laryngeal cancer has yet to be elucidated. Bone marrow cells (including megakaryocytes) dys-function may contribute to altered PDW. PDW is a measure of platelet heterogeneity caused by heterogeneous demarcation of megakarocytes [16]. Recent reports demonstrated several cytokines, such as interleukin-6 (IL-6), granulocytes colony stimulating factor (G-CSF) and macrophage colony stimulating factor (M-CSF), regulate megakaryocytic maturation, platelet production and platelet size [17]. IL-6 promotes tumor angiogenesis, metastasis and metabolism [18]. Furthermore, the cytokines G-CSF and M-CSF that be secreted by tumor cells could stimulate megakaryopoiesis and subsequent thrombopoiesis in cancer [19]. Another possible mechanism is that platelets promote the hypercoagulable state in cancer. Activated platelets create a procoagulant micro-environment that enables the tumor cells to cover themselves with platelets and evade the host immune system [20].

A previous study showed that high platelet-to-lymphocyte ratio predicts poor prognosis in patients with laryngeal carcinoma, suggesting the crucial role of platelets in tumor growth and tumor invasion [21]. Our present study confirmed the key role of activated platelet in laryngeal cancer using a simple platelet parameter. These data are also in line with the current concept that anti-platelet is considered to be a part of cancer adjuvant therapy [2]. Moreover, our findings will provide some promising guidance for clinical management and personalized therapeutics in treating patients with laryngeal cancer.

The study has couple of limitations. First, this was a single-center retrospective study and additional larger validation studies with multiethnic groups are needed to confirm our results. Second, we were unable to explore the exact mechanism of PDW in laryngeal cancer. Third, the patients were composed of Chinese. The application to other ethnic groups still needs further investigation.

In conclusion, higher PDW may serve as a marker of adverse prognosis findings on activated platelets in laryngeal cancer. Further studies are warranted to clarify the exact role of PDW in laryngeal cancer.

## MATERIALS AND METHODS

### Study population

This retrospective study examined the records of 241 consecutive cases with a diagnosis of laryngeal cancer between January 2009 and December 2009 within the Harbin Medical University Cancer Hospital. All patients undergone surgical resection for laryngeal cancer. The pathologic diagnoses of laryngeal cancer were evaluated by pathologists from biopsy reports. None of the patients received preoperative chemotherapy or radiation therapy. Patients were excluded if they had hematological disorders, coronary artery disease, hypertension, diabetes mellitus, and medical treatment with anticoagulant, statins, and acetylic salicylic acid.

Standard demographic and clinicopathological data were collected from the patients’ records in hospital. Survival data were obtained through follow-up. Overall survival (OS) was defined as the interval from the date of diagnosis to death or last follow-up. The median follow-up time was 60 months.

The Institutional Ethics Review Board of the Harbin Medical University Cancer Hospital approved this study prior to commencement of data collection and waived the informed consent requirement because it was a retrospective study.

### Statistical analysis

All statistical analyses were performed using SPSS Statistics version 22.0 (SPSS Inc., Chicago, IL, USA). All continuous data are expressed as means ± SD or medians (interquartile range), and the categorical data are expressed in percentages. The continuous variables were compared with Student's t test or the Mann-Whitney U test, as appropriate, whereas categorical variables were compared with the Chi-square test. A receiver operating characteristic (ROC) curve was generated to find cutoffs of MPV and PDW with optimal diagnostic sensitivity and specificity. The analysis of overall survival was performed by the Kaplan-Meier method and compared using the Log-rank test. To determine prognostic factors, multivariate regression analysis was performed using the Cox proportional hazards model for variables with P < 0.10 in the univariate Cox analyses. Differences were considered significant at P < 0.05.
